# Dynamic Accuracy of GPS Receivers for Use in Health Research: A Novel Method to Assess GPS Accuracy in Real-World Settings

**DOI:** 10.3389/fpubh.2014.00021

**Published:** 2014-03-10

**Authors:** Jasper Schipperijn, Jacqueline Kerr, Scott Duncan, Thomas Madsen, Charlotte Demant Klinker, Jens Troelsen

**Affiliations:** ^1^Research Unit for Active Living, Department of Sport Science and Clinical Biomechanics, University of Southern Denmark, Odense, Denmark; ^2^Department of Family and Preventive Medicine, University of California San Diego, San Diego, CA, USA; ^3^Human Potential Centre, Auckland University of Technology, Auckland, New Zealand

**Keywords:** global positioning system, travel mode, environmental conditions, Qstarz BT-Q1000XT, epoch, validation study, dynamic accuracy

## Abstract

The emergence of portable global positioning system (GPS) receivers over the last 10 years has provided researchers with a means to objectively assess spatial position in free-living conditions. However, the use of GPS in free-living conditions is not without challenges and the aim of this study was to test the dynamic accuracy of a portable GPS device under real-world environmental conditions, for four modes of transport, and using three data collection intervals. We selected four routes on different bearings, passing through a variation of environmental conditions in the City of Copenhagen, Denmark, to test the dynamic accuracy of the Qstarz BT-Q1000XT GPS device. Each route consisted of a walk, bicycle, and vehicle lane in each direction. The actual width of each walking, cycling, and vehicle lane was digitized as accurately as possible using ultra-high-resolution aerial photographs as background. For each trip, we calculated the percentage that actually fell within the lane polygon, and within the 2.5, 5, and 10 m buffers respectively, as well as the mean and median error in meters. Our results showed that 49.6% of all ≈68,000 GPS points fell within 2.5 m of the expected location, 78.7% fell within 10 m and the median error was 2.9 m. The median error during walking trips was 3.9, 2.0 m for bicycle trips, 1.5 m for bus, and 0.5 m for car. The different area types showed considerable variation in the median error: 0.7 m in open areas, 2.6 m in half-open areas, and 5.2 m in urban canyons. The dynamic spatial accuracy of the tested device is not perfect, but we feel that it is within acceptable limits for larger population studies. Longer recording periods, for a larger population are likely to reduce the potentially negative effects of measurement inaccuracy. Furthermore, special care should be taken when the environment in which the study takes place could compromise the GPS signal.

## Introduction

The importance of understanding the environmental context in which health-related behaviors take place is becoming increasingly accepted in behavioral health research [see Ref. (1) for a comprehensive overview of the reviews in this field]. The emergence of portable global positioning system (GPS) receivers over the last 10 years has provided researchers with a means to objectively assess spatial position in free-living conditions. Coupled with other instruments, such as motion sensors (accelerometers), travel diaries, and geographic information systems (GIS), the positional data obtained from GPS can enable the environmental context of health-related behaviors to be elucidated (2). Each year, new GPS receivers are released that improve on previous generations of devices, becoming smaller, cheaper, more reliable, and with extended battery duration. As a consequence, the collection of supplementary contextual information in behavioral health research has become more feasible than ever. GPS has for example been used to investigate the effects of food environments on eating patterns (3), various physical activity related behaviors [see Ref. (4) for a review], independent mobility in youth (5, 6), and the health effects of exposure to pollutants (7, 8). Given the advantages of the objective assessment of environmental exposure, it is likely that the use of GPS in health research will continue to increase.

However, the use of GPS in free-living conditions is not without challenges: a recent review revealed that many studies had significant problems with data loss due to signal drop-outs, loss of battery power, and poor participant compliance (4). Issues with inconsistent GPS signals, such as total signal loss or poor accuracy typically occur due to signal reflection off buildings, or shading by buildings or tree-cover. Signal strength can also be influenced by the availability of GPS satellites in the sky during different times of the day, season, and at different latitudes (lower altitude, northern hemisphere locations typically have the best satellite coverage). The signal strength at a given time and location is expressed as the dilution of precision (DOP), given these limitations, the purpose of this study is to establish the measurement accuracy of GPS receivers under a variety of environmental conditions prior to implementation as a research tool.

Previous research has demonstrated the varying levels of positional accuracy between different GPS receivers and environmental conditions when stationary (9). Conducting a high quality assessment of the static positional accuracy of GPS units is relatively straightforward: GPS units are placed on an official geodetic point for which the exact location is known, with the difference between the recorded position and the actual position of the geodetic point providing an estimate of positional accuracy [see, e.g., Ref. (9)]. Evaluating the positional accuracy of GPS units under dynamic conditions (i.e., free-living) is more challenging as precise data on the “true” route is often not readily available. In many GIS roads are digitized as center-lines with the road-width specified as a category in the attribute-table; i.e., data on the exact width of vehicle lanes, bicycle lanes, and sidewalks are often not available in the GIS. Different methods have been used by various researchers to test the dynamic accuracy of many different GPS devices. Rodriguez et al. (10) used the average location recorded from multiple units of the same device (Garmin Foretrex 201) to assess accuracy under a variety of free-living scenarios. They found that the average distance between each unit and the average of five other identical units was 10.7 ± 11.9 m in open space scenarios and 20.1 ± 21.8 m in clustered development scenarios (10). However, the method used by Rodriguez and colleagues was essentially a test of consistency across identical devices, rather than systematically testing dynamic accuracy under different environmental conditions. The dynamic accuracy of five GPS devices (GlobalSat DG-100 and BT-335, Wintec WBT-201, Visiontac VGPS-900, Qstarz BT-Q1000X) was tested by Wu et al. (11) by digitizing six different routes on a high resolution (1 m) aerial photograph and calculating the percentage of points within 10 and 20 m of the route. Results showed considerable variations by route, mode of travel, and GPS device, with values ranging from 20 to 50% of points falling within 10 m of the route. Each route was traveled twice, and there were considerable differences in accuracy between the two runs (11). The median error varied between GPS devices from 3.5 m for the GlobalSat DG-100 to 5.5 m for the Visiontac VGPS-900; the Qstarz BT-Q1000X had a median error of 4.6 m (11). The study by Wu and colleagues focused on testing the difference between devices and did not systematically test different environments, for different modes of transport. Wieters and colleagues (12) tested the dynamic accuracy of four GPS devices (Garmin Forerunner 205, Garmin Foretrex 201, GlobalSat DG-100, Wintec Easy Showily). Four test persons walked one-time along one pre-defined route and the percentage of recorded data points that fell within five feet of the prescribed course was calculated; more detail on how this was done was not reported in the paper. For the four different GPS devices, the percentage of points that were correctly located on the sidewalk ranged from 57.2 to 76.0% (12). Beekhuizen and colleagues (13) tested dynamic accuracy of two vehicle tracking GPS devices (TracKing Key Pro and the Adapt AD-850), as well as a hiking GPS (Garmin Oregon 550). Their test included assessment of the dynamic accuracy in various modes of transport during commuting. The “true routes” of 12 test persons were mapped as a line on top of a high resolution aerial photograph and the median positional errors compared to these routes were calculated. Each route was traveled twice and the median error was 3.7 m for walking, 2.9 m for biking, 4.8 m for train, 4.9 m for bus, and 3.3 m for car trips (13). There were no significant differences between the three tested devices. In a second phase, spatial accuracy was tested during a walking trip under six different environmental conditions and repeated 10 times. There were considerable differences and the overall median error ranged from 2.2 m for a relatively open residential area to a median error of 7.1 m for a commercial high-rise area (13).

The results from these four studies are difficult to compare directly to each other as they used different methods and devices. Furthermore, none of these studies reported studying the potential interaction of various modes of transport within different environmental conditions. Since participants in free-living studies are likely to spend a significant amount of time in dynamic movement, and transportation mode is an important correlate of health, it is vital to know the dynamic accuracy during different modes of transport under a variety of environmental conditions. Further, if researchers wish to study use of existing facilities such as bicycling lanes, or environmental changes such as new sidewalks and pedestrian crossings, it is important to understand whether tracking behavior at this level of accuracy is possible under different environmental conditions.

Finally, the potential effects of changing the data collection interval on positional accuracy are poorly understood. It could be that collecting data more often (i.e., with a shorter epoch) improves the overall positional accuracy by being able to track a route more precisely, e.g., in situations with many changes of direction the number of “cut corners” might be reduced. Frequent pinging to the satellite, however, in high interference environments could result in more missing or misplaced data. Further, the advantage of collecting more points might be outweighed by reduction in the total data collection period due to the available device memory filling-up more quickly without improving spatial accuracy.

The aim of this study was to test the dynamic accuracy of a portable GPS device under real-world environmental conditions, for four modes of transport, using three data collection intervals.

## Materials and Methods

### Instruments

We selected four routes in the City of Copenhagen, Denmark, to test the dynamic accuracy of the Qstarz BT-Q1000XT GPS device. This model was selected not only for its common usage in previous and current research, but also for its relatively high accuracy under various environmental conditions, good signal acquisition time, data storage, and battery life (9). Two research assistants followed a predetermined protocol of walking, cycling, driving, and bussing on all routes, in both directions, while wearing three GPS devices, each set to record data at a different data collection interval (epoch).

### Test routes

**Figure 1:**
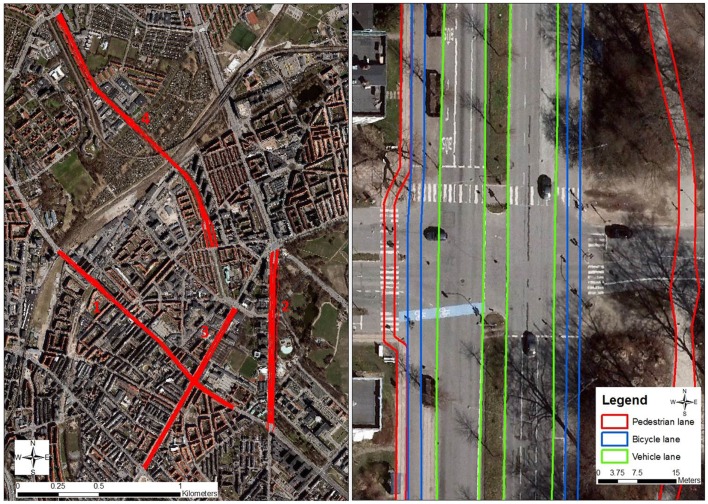
**Location of the four study routes (left) and example of the detailed digitization of vehicle, bicycle, and pedestrian lanes (right)**.

The four test routes were on different bearings, passing through a variation of environmental conditions. We selected the routes on different bearings as we hypothesized that the effect of buildings shading for GPS signal reception would vary according to bearing. In Denmark, the position of satellites in the sky is predominantly southwards, which, in theory, should favor satellite reception on more north–south directed routes, whereas east–west directed routes, especially the south-side of route, are more likely to be shaded from good satellite reception by adjacent buildings. We furthermore made sure that each route consisted of separate pavements, bicycle lanes and vehicle lanes in both directions, and that public busses were running along each route. Each route consisted of a walk, bicycle, and vehicle lane in each direction, in total six different lanes per route (see Figure 1, left). All 24 lanes were manually digitized as polygons in ArcGIS 10.1 using ultra-high-resolution (10 cm pixels) aerial photographs as background. The actual width of each walking, cycling, and vehicle lane was digitized as accurately as possible. Euclidean (as the crow flies) buffers of 2.5, 5, and 10 m were created for each lane (see Figure 1, right). To classify the environment along the routes, all buildings along the route were buffered with 25 m, and the environment was classified as open if there were no buildings within 25 m of the route, half-open if there were buildings within 25 m on one side of the route, and an urban canyon if there were buildings closer than 25 m on both sides. Finally, along one of the routes, there was a smaller area where the environment was classified as tree-covered since there were trees next to the lanes and the tree canopy was fully covering the bicycle and pedestrian lanes. Route 1 was 1.4 km long relatively narrow road with five to six story buildings directly adjacent to the route for the most part, and on a bearing varying between 332° and 334°. Route 2 was 1.1 km long relatively wide road with a large park on one side of the road, with part of road under tree-cover, and scattered six to seven story buildings close to the road on the other side. Route 2 ran almost exactly north–south with a bearing of 1°. Route 3 was 1.2 km long, with some sections with five to six story buildings directly adjacent, whereas other sections only had buildings on one side. The bearing for route 3 was 22°. Route 4 was 1.8 km long on a bearing varying between 324° and 338°. For the most part, route 4 did not have buildings directly adjacent to the road; they were typically placed 10–25 m from the road.

### Data collection

Two research assistants followed a predetermined protocol of walking, cycling, driving, and bussing on all routes, in both directions, recording data on 300 trips. The date and exact start and end times of each trip were recorded by the research assistants, using a digital watch that was synchronized to the GPS satellite time. To be able to determine if GPS data recording intervals influenced the positional accuracy, data were collected simultaneously at three different epochs, 5, 15, and 30 s respectively. The three GPS devices were worn on one elastic belt around the waist, covered by clothing. Using the open source bt747 GPS software (www.bt747.org), the GPS units were set to collect longitude and latitude, elevation, speed, the number of satellites used and in view, the satellite number of the used satellites, and the DOP values, for the horizontal (HDOP), vertical (VDOP), and positional, i.e., 3-dimensional (PDOP) DOP. DOP values express the expected uncertainty associated with the alignment of available satellites at a certain time and location; a DOP value <1 is considered ideal, one to two is excellent, whereas a DOP value of more than 10 indicates unfavorable satellite geometry.

### Data processing

After each trip the data were downloaded using the open source bt747 GPS software and not processed further before analyses (i.e., potential outliers or otherwise clearly faulty data was not removed). Based on the date–time log for start and end times of each trip, all GPS points belonging to each trip were identified. Using the spatial join function in ArcGIS, we identified the GPS points for each trip that fell within the corresponding lane polygon, or within the 2.5, 5, and 10 m buffers of that lane polygon. For each trip, we calculated the percentage that actually fell within the lane polygon, and within the 2.5, 5, and 10 m buffers respectively, as well as the mean and median error in meters. We differentiated results for each of the four trip modes, for each of the three data collection epochs, and for each of the four environmental types.

## Results

### Overall dynamic spatial accuracy

The results in Table 1 show that the overall median distance error from the lane traveled in was 2.9 m (IQR 0.4–8.4 m), and that 78.9% of the GPS points fell within 10 m of the actual lane and 46.9% within 2.5 m. The median error of the GPS receiver during walking trips was 3.9, 2.0 m for bicycle trips, 1.5 m for bus, and 0.5 m for car. The 10-m dynamic accuracy was 73.5% (walk), 86.8% (bicycle), 84.9% (bus), and 89.3% (car), respectively. Across modes of transport and type of area, the median error for the three data collection epochs was the same, and also the IQR’s and percent of points within 2.5 and 10 m from the lane was similar. The four different types of areas along the route showed considerable variation in the median error: 0.7 m in open areas, 2.6 m in half-open areas, and 5.2 m in urban canyons. Points on the small tree-covered section had a median error of 1.0 m. There were also clear differences between the four routes, with a 0.7-m median error for route 2, 3.5 and 3.7 m for routes 3 and 4, respectively, and 4.5 m for route 1.

**Table 1:** **Dynamic spatial accuracy in percent of points and mean and median errors in meters, overall as well as divided by trip mode, epoch, area type, and route**.

		*n*	% Of points			Distance from lane in meters				
			Within lane	<2.5 m Outside of lane	<10 m Outside of lane	Mean	SD	Median	IQR	
Trip mode	Walking	40,154	13.2	40.0	73.5	8.2	11.8	3.9	1.0	10.7
	Bicycle	13,777	22.3	55.1	86.8	5.0	8.7	2.0	0.2	5.5
	Bus	11,656	37.1	57.3	84.9	5.7	12.0	1.5	0.0	6.2
	Car	2338	45.6	64.4	89.3	4.1	9.5	0.5	0.0	4.4
Epoch	5 s	45,008	20.3	47.0	79.3	6.8	10.7	2.9	0.4	8.1
	15 s	15,394	20.5	46.7	77.5	7.4	11.7	2.9	0.3	8.9
	30 s	7523	19.8	46.6	77.5	7.7	13.6	2.9	0.4	8.8
Area type	Open	5827	36.3	71.9	88.8	4.3	9.6	0.7	0.0	2.9
	Half-open	36,834	20.8	49.1	82.2	5.9	9.3	2.6	0.3	7.0
	Urban canyon	20,171	14.3	33.3	69.6	9.3	12.4	5.2	1.3	12.2
	Tree-covered	2656	24.3	74.2	97.9	1.9	3.0	1.0	0.0	2.6
Route	1	19,751	15.5	36.6	71.7	9.0	13.1	4.5	1.0	11.5
	2	13,801	34.7	76.0	92.8	3.6	10.3	0.7	0.0	2.4
	3	14,958	18.0	42.7	76.0	7.6	11.1	3.5	0.6	9.6
	4	19,415	16.5	39.9	78.0	6.9	9.4	3.7	0.9	8.8
Overall		67,925	20.3	46.9	78.7	7.0	11.3	2.9	0.4	8.4

### Variation by area type and trip mode

As can be seen in Table 2, the GPS performed poorest for walking trips in urban canyons (with lots of five to six story directly adjacent buildings) with a median error of 6.7 m. Walking lanes were typical directly adjacent to buildings, and as low as 61.9% of all GPS points were within 10 m of the walking lanes. GPS data collected during car trips within urban canyons had a surprisingly high 10 m accuracy, 88.4%, only slightly less than the 89.5% in open areas. The median error for car trips in urban canyons was 1.5 m.

**Table 2:** **Dynamic spatial accuracy in percent of points and mean and median errors in meters, for four modes of transport within three area types**.

Area type	Trip mode	*n*	% Of points			Distance from lane in meters				
			Within lane	<2.5 m Outside of lane	<10 m Outside of lane	Mean	SD	Median	IQR	
Open	Walking	2592	26.6	43.6	85.9	5.1	10.2	1.0	0.0	3.3
	Bicycle	1559	37.8	35.9	91.4	3.4	7.7	0.6	0.0	2.7
	Bus	1399	50.3	23.0	91.2	3.8	10.0	0.0	0.0	2.8
	Car	277	48.0	22.4	89.5	4.3	9.8	0.1	0.0	3.0
Half-open	Walking	22,910	13.4	27.6	77.0	7.2	10.1	3.6	0.9	9.2
	Bicycle	7620	24.7	36.5	91.3	3.9	7.3	1.6	0.0	4.1
	Bus	5410	40.4	22.0	89.7	4.0	8.0	1.0	0.0	4.8
	Car	894	58.7	15.8	92.2	2.5	5.4	0.0	0.0	2.7
Urban canyon	Walking	11,124	8.0	18.3	61.9	11.5	14.0	6.7	2.3	15.7
	Bicycle	3969	13.1	22.1	76.1	7.7	10.7	4.4	1.4	9.6
	Bus	3959	27.0	17.5	79.1	6.1	8.2	3.4	0.0	8.7
	Car	1119	35.7	21.1	88.4	4.2	7.0	1.5	0.0	5.3

### Variation by area type, trip mode and data collection epoch

Looking at the results in Table 3 for the dynamic spatial accuracy of the GPS devices in different data collection epoch, divided by area types and by trip modes, it seems that the differences are small. In urban canyons however, the shortest epoch seems to perform slightly better for all trip modes; the median error is 0.2–0.4 m lower at a 5-s data collection epoch than it is at a 15-s epoch. The 15-s epoch does not consistently perform better than the 30-s epoch, but slight improvements can be seen in some conditions, e.g., for walking and bicycling in urban canyons the median error is lower at 15 s compared to a 30-s epoch.

**Table 3:** **Dynamic spatial accuracy in percent of points and mean and median errors in meters, for three different data collection epochs, within four modes of transport, within three area types**.

Area type	Trip mode	Epoch (s)	*n*	% Of points			Distance from lane in meters				
				Within lane	<2.5 m Outside of lane	<10 m Outside of lane	Mean	SD	Median	IQR	
Open	Walking	5	1673	26.5	44.6	87.3	4.6	9.2	1.0	0.0	3.1
		15	624	28.2	40.4	84.0	5.7	11.0	1.0	0.0	3.4
		30	295	23.7	44.4	81.7	6.5	13.3	1.1	0.0	4.2
	Bicycle	5	1013	38.7	36.4	91.7	3.1	7.0	0.6	0.0	2.5
		15	364	36.3	36.3	90.1	3.9	8.7	0.6	0.0	2.8
		30	182	35.7	31.9	92.3	4.0	9.2	0.9	0.0	3.0
	Bus	5	910	51.9	21.5	90.4	3.8	10.0	0.0	0.0	2.7
		15	326	50.0	25.5	93.3	3.8	11.1	0.0	0.0	2.4
		30	163	41.7	26.4	91.4	3.5	7.9	0.7	0.0	3.4
	Car	5	171	50.9	21.6	92.4	3.1	7.4	0.0	0.0	2.7
		15	68	52.9	22.1	91.2	3.5	8.4	0.0	0.0	2.5
		30	38	26.3	26.3	73.7	11.1	17.1	1.9	0.0	10.9
Half-open	Walking	5	15,271	13.2	27.0	77.6	7.0	9.6	3.7	1.0	9.0
		15	5130	14.1	28.7	75.2	7.8	11.0	3.4	0.8	9.9
		30	2509	13.2	28.9	76.5	7.4	10.7	3.5	0.8	9.4
	Bicycle	5	5069	25.7	37.8	92.5	3.6	7.0	1.5	0.0	3.8
		15	1675	22.9	33.6	87.3	4.8	8.3	2.0	0.1	5.4
		30	876	22.1	35.3	91.3	3.9	6.7	1.9	0.2	4.5
	Bus	5	3531	40.5	22.3	89.9	3.9	7.9	0.9	0.0	4.7
		15	1246	40.1	21.2	89.4	4.3	8.6	1.0	0.0	5.1
		30	633	40.4	21.6	89.1	4.0	7.4	1.0	0.0	5.1
	Car	5	578	62.5	13.8	94.3	2.2	5.2	0.0	0.0	2.1
		15	219	54.3	21.0	90.4	2.5	4.7	0.0	0.0	2.5
		30	97	46.4	15.5	83.5	4.4	7.2	0.4	0.0	5.6
Urban canyon	Walking	5	7430	8.0	18.6	62.1	11.5	13.9	6.6	2.3	15.7
		15	2533	8.3	17.6	62.9	11.1	12.8	6.8	2.3	15.2
		30	1161	7.3	18.3	58.5	12.7	16.7	7.2	2.4	16.5
	Bicycle	5	2663	13.2	22.9	76.4	7.3	9.7	4.3	1.3	9.6
		15	875	12.9	19.3	76.5	8.0	11.1	4.7	1.6	9.5
		30	431	13.0	22.5	73.8	9.2	15.3	4.8	1.4	10.6
	Bus	5	2626	27.2	17.3	80.6	5.8	7.7	3.3	0.0	8.1
		15	908	27.0	16.9	76.8	6.7	9.6	3.7	0.0	9.6
		30	425	26.1	19.5	75.3	6.4	8.4	3.6	0.0	9.9
	Car	5	735	36.1	21.6	89.0	4.0	6.5	1.3	0.0	4.8
		15	255	38.0	20.4	87.8	3.9	6.1	1.6	0.0	6.0
		30	129	28.7	19.4	86.0	5.9	10.6	2.7	0.0	5.9

### Variation by mode and route

The data presented in Table 4 shows differences between the half-open sections of the four routes, for walking, bicycling, and bus trips. Clear differences between the routes can be seen, even though the points included in Table 4 were all within half-open environments. Across the three trip modes, the GPS’s performed best along route 2, with a north–south bearing (median error 0.0–0.9 m). For walking the GPS performed poorest on route 4, with a northwest–southeast bearing (median error 5.4 m) while it did poorest for cycling on route 1, also on a northwest–southeast bearing (median error 4.5 m), and for bus trips on route 3, types on a northeast–southwest bearing (median error 2.4 m).

**Table 4:** **Dynamic spatial accuracy in percent of points and mean and median errors in meters, for four different routes, within three modes of transport, within half-open areas**.

Half-open	Route	*n*	% Of points			Distance from lane in meters				
Trip mode			Within lane	<2.5 m Outside of lane	<10 m Outside of lane	Mean	SD	Median	IQR	
Walking	1	3776	11.5	24.2	69.4	10.0	15.0	4.7	1.3	13.0
	2	4179	29.0	48.4	95.5	2.1	4.5	0.9	0.0	2.3
	3	3447	15.5	33.7	82.5	6.0	9.2	2.6	0.5	6.7
	4	11,508	7.7	19.3	71.1	8.5	9.1	5.4	2.3	11.4
Bicycle	1	1339	11.2	23.2	76.8	7.5	9.4	4.5	1.4	9.3
	2	1291	35.2	47.8	95.3	2.8	8.2	0.6	0.0	1.9
	3	1099	22.7	32.2	83.3	5.8	9.7	2.0	0.2	6.0
	4	3891	26.3	38.7	97.1	2.5	4.1	1.4	0.0	3.5
Bus	1	1094	35.9	20.5	83.1	5.2	9.3	1.8	0.0	6.5
	2	865	58.6	19.5	98.5	1.5	2.6	0.0	0.0	1.7
	3	1211	29.8	20.8	77.5	7.5	12.4	2.4	0.0	9.0
	4	2240	41.3	24.2	96.2	2.5	3.8	0.8	0.0	4.0

The HDOP values (data not shown) for 76.4% of the data points were under 1, which is considered ideal, and 95.2% had a value lower than 2, which is considered excellent. The highest HDOP value (6.2) was recorded during one walking trip along route 2, but the median error or percentage of points within the lane during this trip were similar to those of other walking trips along route 2 (data not shown).

## Discussion

The aim of this study was to test the dynamic accuracy of a high performing GPS device that many researchers in public health are employing (Qstarz Q1000XT portable GPS receiver) under different real-world environmental conditions, for four modes of transport, using three data collection epochs. Our results showed that almost half (49.6%) of all ≈68,000 recorded GPS points fell within 2.5 m of the expected location, and 78.7% fell within 10 m. The median error was 2.9 m. There were differences by trip mode, area type, and route, while the three data collection epochs had the same median error (2.9 m for all epochs). The median error of the GPS receiver during walking trips was 3.9, 2.0 m for bicycle trips, 1.5 m for bus, and 0.5 m for car. The four different types of areas showed considerable variation in the median error: 0.7 m in open areas, 2.6 m in half-open areas, and 5.2 m in urban canyons. There were also clear differences between the four routes, with a 0.7-m median error for route 2, 3.5, and 3.7 m for routes 3 and 4, respectively, and 4.5 m for route 1. In practice our results indicate that care should be taken when a high spatial accuracy is required. For example, in study that tries to determine the use of a new playground element, a median error of 5 m under dense urban conditions could mean that a large percentage of points that fall “on” the playground element might in reality be “on” the adjacent element, or vice versa, which could easily lead to wrong conclusions.

Comparing our results to those of Beekhuizen et al. (13), the median error of walking was comparable (3.9 m in our study versus 3.7 m in theirs), while the median errors for cycling, car, and bus trips were smaller in the present study (cycling 2.9 versus 2.0 m; bus 4.9 versus 1.5 m; and car 3.3 versus 0.5 m). Roughly 85% of all errors in the Dutch study were <10 m (13), which is slightly better than the 78.7% in our study. This could be due to the fact that the data was gathered during commuting trips that were located outside dense urban areas (where interference is a problem) to a much larger extent than the present study.

Beekhuizen et al. (13) reported median errors for walking trips in a high-rise commercial area (median error 7.1 m), which is similar to our median error recorded during walking trips in urban canyons (6.7 m). Our finding that there is an interaction between environment and mode of transport was, as far as we know, not reported by other researchers. The accuracy of GPS data collected during car trips within urban canyons was surprisingly high, with a 10-m accuracy of 88.4%, and only slightly less than in open areas (89.5%). The median error for car trips in urban canyons was only 1.5 m; however, this could partly be due to the fact that sidewalks and bicycle paths were typically less wide than the vehicle lanes and considerably closer to buildings. In some parts of the route, the vehicle lane could be as wide as 12 m, which likely increased the chance of the GPS point falling within the lane polygon. This does not diminish the fact that for car and bus trips in real-world settings, the Qstarz GPS device has a surprisingly good spatial accuracy, also in difficult environmental conditions. Nonetheless, our finding also implies that care has to be taken when studying walking and/or cycling behavior in dense urban environments. As walking and cycling lanes are typically located closer to buildings and much narrower than vehicle lanes, the spatial accuracy can be compromised. Furthermore, we also found differences between routes on different bearings, within similar environmental conditions. These differences are likely explained by a different degree of shading by buildings depending on their positions in relation to the observed route. In practice, this means that the dynamic error will differ depending on the angle toward the available satellites, which will differ during the day.

### Strengths and weaknesses

This study is, to our knowledge, the largest and most rigid test of dynamic GPS accuracy conducted to date. Earlier studies (10–13) have also assessed the dynamic accuracy, but with different methods and smaller samples, and without looking specifically at different modes of transport in varying environmental conditions, or at different data collection epochs. Digitizing all traffic lanes individually on top of high resolution aerial photographs led to highly detailed route maps that were used as the “true” route.

This study demonstrated that it might be important to test routes with narrower vehicle lanes, although such streets may not have separate bicycle lanes or public buses along the same route. In addition, all data were collected in late fall, winter, and early spring, and most trees had little or no leaves, which could have improved the average satellite reception on two of the four routes. A study comparing the impact of tree-cover across the seasons would be an important next step.

Furthermore, there is a range of other factors not included in this study that could influence the positional accuracy. GPS receivers require a direct line of sight with at least four satellites to determine a spatial position by means of triangulation. In obstructed conditions, such as indoors or underneath a tree canopy, or in the “shade” of a tall buildings, signal inconsistencies arising from limited satellite visibility, and/or reflection of signal off nearby buildings or objects (multipath effect) can result in significant positional error (7, 9, 10, 14). In particular the presence of water reservoirs, metal, or other reflecting surfaces tends to result in so-called multipath effect; i.e., the GPS does not only receive signals directly from the satellites, but also signals reflected from such surfaces.

Other potential sources of GPS inaccuracy include timing errors, orbital errors, and atmospheric disturbances (9). The Qstarz Q1000XT is equipped with differential GPS (DGPS) capability – a system that broadcasts corrections from ground-based reference stations to surrounding GPS receivers in real time – which can reduce this type of errors.

### Recommendations for using GPS in public health studies

Based on our findings, the positional accuracy of the Qstarz Q1000XT GPS receiver in dynamic and varied conditions is acceptable for use in larger population studies, especially with relatively long data collection periods (7 days or more). For studies where participants live in, or travel through, dense urban areas we would recommend conducting a dynamic accuracy test similar to the one presented here to determine if the accuracy achieved is acceptable in relation to the research question. Based on our findings, we would also recommend that researchers interested in recording behavior in specific dense urban locations (e.g., recording the use of new pedestrian or bicycle facilities, or other challenging environments such as schoolyards) to field test GPS accuracy in those specific locations, during different times of the day to determine if the error is acceptable for their study. In future, it will be useful to also test the dynamic accuracy of other GPS units to be used for public health studies.

## Conclusion

Our results showed that almost half (49.6%) of all ≈68,000 GPS points recorded with the Qstarz Q1000XT GPS units fell within 2.5 m of the expected location, 78.7% fell within 10 m and the median error was 2.9 m. The median error of the GPS receiver during walking trips was 3.9, 2.0 m for bicycle trips, 1.5 m for bus and 0.5 m for car. The four different types of areas showed considerable variation in the median error: 0.7 m in open areas, 2.6 m in half-open areas and 5.2 m in urban canyons.

The dynamic spatial accuracy of this device is not perfect, but we feel that it within acceptable limits for larger population studies. However, it is important for researchers to consider when deciding on sample sizes and recording periods. Longer recording periods for a larger population are likely to reduce the potentially negative effects of measurement inaccuracy. Furthermore, special care should be taken when the environment in which the study takes place could compromise the GPS signal (i.e., very dense urban locations).

## Conflict of Interest Statement

The authors declare that the research was conducted in the absence of any commercial or financial relationships that could be construed as a potential conflict of interest.
